# Incidence and Outcomes of Invasive Aspergillosis in Hospitalized Patients with Pancreatic Transplantation: A Nationwide Population-Based Analysis

**DOI:** 10.3390/microorganisms14030669

**Published:** 2026-03-16

**Authors:** Aditya Sharma, Marc Piper, Rahul Maheshwari, Ayman O. Soubani

**Affiliations:** 1 Department of Internal Medicine, Wayne State University School of Medicine, Detroit 48201, MI, USA; hw8777@wayne.edu; 2 Division of Gastroenterology and Hepatology, Trinity Health Ann Arbor Hospital, Ann Arbor 48197, MI, USA; piperm@hurongastro.com; 3 Division of Hepatocellular Carcinoma, Piedmont Transplant Institute, Atlanta 30309, GA, USA; rahul.maheshwari@piedmont.org; 4 Division of Pulmonary, Critical Care and Sleep Medicine, Wayne State University School of Medicine, Detroit 48201, MI, USA

**Keywords:** invasive aspergillosis, pancreatic transplant, fungal infection, invasive mechanical ventilation

## Abstract

Background: Invasive Aspergillosis (IA) is a rare but life-threatening fungal infection in immunocompromised hosts, including solid organ transplant (SOT) recipients. While extensively studied in other SOT populations, data on IA in pancreas transplant (PT) recipients are limited. Earlier studies reported mortality rates nearing 100%, whereas more recent data show that 12-week mortality still exceeds 20% despite improvements in antifungal therapy. Current prophylaxis strategies for PT recipients mainly focus on *Candida* species, and there are no clear, standardized recommendations for *Aspergillus* prevention. Given the paucity of focused data, the epidemiology, clinical characteristics, and outcomes of IA in PT recipients are not well defined. This study aimed to assess the incidence, clinical characteristics, and outcomes of IA among hospitalized PT patients using a nationally representative dataset. Methods: We conducted a descriptive analysis using the National Inpatient Sample (NIS) from 2016 to 2020. PT admissions were identified using International Classification of Diseases, Tenth Revision (ICD 10) codes for transplant status and procedures. IA was defined using validated ICD 10 codes. Baseline demographics, hospital characteristics, comorbidities, and outcomes, including sepsis, acute kidney injury (AKI), acute respiratory failure (ARF), invasive mechanical ventilation (IMV), all-cause in-hospital mortality, length of stay, and total hospitalization costs and charges were compared between PT admissions with and without IA. National estimates were calculated using discharge weights, and comparisons were performed using the chi-square test and adjusted Wald test. Multivariable analysis was performed to identify predictors of all-cause in-hospital mortality among PT admissions complicated by IA. Two-sided *p* values < 0.05 were considered statistically significant. Results: Between 2016 and 2020, 65,980 PT-related hospitalizations were identified, of which 250 (0.4%) had IA. PT admissions complicated by IA were more commonly aged 41 to 60 years (59% vs. 46%, *p* = 0.012) and were less likely to have a Charlson Comorbidity Index greater than 3 (54% vs. 68.6%, *p* < 0.001) compared with PT hospitalizations without IA. The PT with the IA cohort had higher rates of sepsis (100% vs. 46.1%, *p* < 0.001), AKI (60% vs. 36.7%, *p* < 0.001), ARF (28% vs. 9.4%, *p* < 0.001), and IMV use (18% vs. 4%, *p* < 0.001) compared with the PT without the IA cohort. Among PT hospitalizations with IA, IMV use was independently associated with higher all-cause in-hospital mortality (adjusted odds ratio 48.777, *p* = 0.009). Overall, in-hospital mortality was significantly higher in PT hospitalizations with IA compared with those without IA (12% vs. 2%, *p* < 0.001). Mean length of stay was longer (24.86 vs. 6.13 days, *p* < 0.001), and total charges ($378,494 vs. $94,938, *p* < 0.001), and total costs ($93,019 vs. $24,463, *p* = 0.023) were significantly higher compared with PT hospitalizations without IA. Conclusion: Although rare, IA in PT recipients is associated with higher rates of sepsis, AKI, ARF, venous thromboembolism, prolonged hospitalization, increased mortality, and greater healthcare utilization. Despite the inherent limitations of administrative datasets, this nationally representative analysis highlights the substantial clinical and economic burden of IA in this high-risk population. These findings emphasize the need for targeted surveillance, early diagnosis, and evidence-based antifungal strategies in this vulnerable population.

## 1. Introduction

Invasive aspergillosis (IA) is a severe opportunistic fungal infection associated with high mortality, particularly among immunocompromised individuals [1,2,3]. Its association with solid organ transplant (SOT) recipients is well recognized, largely due to the intense and prolonged immunosuppression needed to maintain graft function and prevent rejection, which increases vulnerability to opportunistic infections such as IA. Earlier studies reported mortality rates approaching 100%, while more recent data indicate that 12-week mortality still exceeds 20% [4,5,6]. The outcomes remain poor due to delays in diagnosis, nonspecific clinical presentation, and the rapid progression of invasive disease, which can result in respiratory failure and multiorgan dysfunction. Although antifungal prophylaxis guidelines exist for *Candida* species in pancreas transplant (PT) recipients, there are currently no standardized recommendations specifically targeting *Aspergillus* prophylaxis [6,7].

Compared with other SOT populations, including lung and liver transplant recipients, data on IA in PT recipients remain especially limited. The epidemiology, risk factors, and clinical outcomes in this subgroup are not well characterized. In this study, we aimed to address this knowledge gap by providing a comprehensive descriptive analysis of the burden, complications, and outcomes associated with IA in PT recipients using a nationally representative dataset. These findings may help inform future risk stratification and guide preventive strategies for invasive fungal infections in this underrecognized, high-risk population.

## 2. Materials and Methods

### 2.1. Data Source

To address this knowledge gap, we conducted the first national-level descriptive analysis evaluating IA among PT recipients using the National Inpatient Sample (NIS) database. The NIS is well-suited for studying rare conditions due to its large, population-based design [8].

This retrospective descriptive cohort study utilized the NIS database from 2016 through 2020. Given the release lag of the NIS, the 2021 dataset was not available at the time this analysis was conducted and was therefore not included. The NIS is the largest publicly available all-payer inpatient database in the United States. It was developed by the Healthcare Cost and Utilization Project (HCUP) and is sponsored by the Agency for Healthcare Research and Quality. The NIS captures a 20% stratified sample of hospital discharges from U.S. community hospitals and represents more than 35 million hospitalizations annually.

Each observation in the database represents a single hospitalization and includes one primary diagnosis, up to 39 secondary diagnoses, and up to 25 procedure codes. Diagnoses are coded using the International Classification of Diseases, Tenth Revision, Clinical Modification (ICD 10 CM), and procedures are coded using the International Classification of Diseases, Tenth Revision, Procedure Coding System (ICD 10 PCS).

### 2.2. Study Population

PT-related admissions were identified using both ICD 10 CM code Z94.83 (pancreas transplant status) and ICD 10 PCS procedure codes corresponding to pancreas transplantation, including allogeneic open approach (0FYG0Z0), syngeneic open approach (0FYG0Z1), and zooplastic open approach (0FYG0Z2). IA was identified using ICD 10 CM codes B44.0 through B44.9, B48.8, and B49.2.

### 2.3. Baseline Variables and Comparison Groups

Baseline variables included demographic characteristics such as age, sex, median household income quartile, and race, categorized as White, Black, Hispanic, and Other (including Asian or Pacific Islander and Native American). Comorbid conditions were identified using ICD 10 CM codes, and they included bronchiectasis, cerebrovascular disease, chronic obstructive pulmonary disease, coronary artery disease, dyslipidemia, tobacco use disorder, hypertension, obesity, and venous thromboembolism (including both pulmonary embolism and deep vein thrombosis). Overall comorbidity burden was also assessed using the Charlson Comorbidity Index (CCI).

In-hospital complications included acute kidney injury (AKI), acute respiratory failure (ARF), and sepsis. The utilization of invasive mechanical ventilation (IMV) was also evaluated. Hospital-level characteristics included teaching status and primary payer. Additional outcomes analyzed included length of stay, total hospitalization costs, total hospitalization charges, and all-cause in-hospital mortality. Baseline characteristics and outcomes were compared between PT-related hospitalizations with and without IA.

### 2.4. Statistical Analysis

National estimates were calculated using HCUP-recommended discharge weights to account for the complex survey design of the NIS [8]. Categorical variables were compared using the chi-square (χ^2^) test, and continuous variables were analyzed using the adjusted Wald test. Results are reported as percentages for categorical variables and as means with standard deviations for continuous variables. Two-sided *p* values < 0.05 were considered statistically significant.

Weighted data were then used to perform adjusted analyses to identify predictors of in-hospital mortality among PT admissions complicated by IA. First, univariable logistic regression was performed to evaluate demographic characteristics, comorbid conditions, and in-hospital complications. Variables with a *p* value < 0.20 in univariable analysis, along with clinically relevant variables such as sex, age, and race, were considered for inclusion in the multivariable logistic regression model. Multivariable logistic regression was subsequently performed to identify independent predictors of all-cause in-hospital mortality among PT recipients with IA. Results are reported as adjusted odds ratios (aORs) with corresponding 95% confidence intervals and *p* values. All statistical analyses were conducted using STAT software version 15.1 (StataCorp, College Station, TX, USA).

As this study used publicly available, de-identified data, it was exempt from institutional review board approval.

## 3. Results

Between the years 2016 and 2020, we identified 65,980 hospitalizations involving either a history of PT or a PT procedure, as defined by the corresponding ICD 10 diagnosis or procedure codes. Of these, 4695 hospitalizations included an active PT procedure. IA was documented in 250 hospitalizations, representing 0.4% of all PT-related admissions during the study period (Table 1).

Among PT hospitalizations complicated by IA, 59% of admissions were aged 41 to 60 years compared with 46% among PT hospitalizations without IA. In contrast, 18.2% of IA-related admissions were aged 61 to 80 years compared with 36% among PT hospitalizations without IA (*p* = 0.012). PT admissions complicated by IA had a significantly lower proportion of hospitalizations with a high comorbidity burden, defined as a CCI greater than three, than PT admissions without IA (54% vs. 68.6%, *p* < 0.001). Conversely, a CCI of ≤1 was more common among IA admissions (20.0% vs. 5.6%), while rates were similar for scores of 2–3 (26.0% vs. 25.9%) (*p* < 0.001).

Pulmonary comorbidities were significantly more frequent in the IA group, including bronchiectasis (4% vs. 0.3%; *p* < 0.001) and chronic obstructive pulmonary disease (18% vs. 5%; *p* < 0.001). Venous thromboembolism, including both deep vein thrombosis and pulmonary embolism, was observed three times more frequently among IA admissions compared with PT admissions without IA (20% vs. 6%; *p* < 0.001). However, other cardiovascular comorbidities, including cerebrovascular disease, coronary artery disease, dyslipidemia, hypertension, and obesity, did not differ significantly between PT admissions with and without IA (Table 1).

All IA admissions were complicated by sepsis, compared with 46.1% of PT admissions without IA (*p* < 0.001). Other in-hospital complications, including AKI (60% vs. 36.7%, *p* < 0.001) and ARF (28% vs. 9.4%, *p* < 0.001), were significantly more common in the IA cohort compared with PT admissions without IA. The requirement for IMV was significantly higher among PT admissions complicated by IA compared with PT admissions without IA (18% vs. 4%, *p* < 0.001). On multivariable logistic regression analysis, after adjusting for hospital-level and patient-level factors, the need for IMV was independently associated with increased odds of all-cause in-hospital mortality among PT admissions complicated by IA (adjusted odds ratio 48.78, 95% confidence interval 2.588–919.360, *p* = 0.009). Other clinically relevant factors, including age, race, and sex, were not found to be statistically significant predictors (Table 2).

Hospital length of stay differed significantly between groups (*p* < 0.001). Among PT admissions without IA, 87.4% had a length of stay ≤10 days compared with 46% of IA admissions. Stays of 11–20 days occurred in 9.2% of non-IA admissions compared with 24% of IA admissions. Similarly, prolonged hospitalizations of ≥21 days were observed in 3.4% of non-IA admissions compared with 30% of IA admissions. Disposition at discharge differed significantly between groups (*p* < 0.001). Routine discharge was less frequent among PT admissions complicated by IA compared with those without IA (40.9% vs. 67.8%). Transfers to a short-term hospital were more common in the IA cohort (9.1% vs. 3.6%). Similarly, transfers to another facility, including skilled nursing facilities, intermediate care facilities, or other healthcare facilities, occurred more frequently among IA admissions (25% vs. 9.6%). Discharge with home health services was also higher in the IA group (22.7% vs. 17.7%). A small proportion of admissions in both groups left against medical advice (Table 1).

AllAll cause in hospital mortality was sixfold higher among PT recipients with IA compared with those without IA (12% vs. 2%; *p* < 0.001). Mean length of stay was significantly longer in IA-related hospitalizations compared with PT hospitalizations without IA (24.86 days vs. 6.13 days; *p* < 0.001). Additionally, total hospitalization charges were approximately fourfold higher in IA-related hospitalizations compared with PT hospitalizations without IA ($378,494 vs. $94,938; *p* < 0.001). Similarly, total hospitalization costs were significantly higher in the IA cohort ($93,019 vs. $24,463; *p* = 0.023) (Table 3).

## 4. Discussion

In this nationally representative study of PT recipients from 2016 to 2020, IA was identified in 0.4% of all PT-related hospitalizations. This admission-level frequency is lower than the 4.8% incidence reported in the 2001 to 2006 TRANSNET study [9]. This difference likely reflects temporal improvements in perioperative management, refinements in immunosuppressive protocols, and broader adoption of infection prevention strategies over the past two decades. In addition, these estimates should be interpreted in light of methodological differences, as the TRANSNET study reported patient-level incidence, whereas our analysis is based on hospitalization-level data. Compared with other solid organ transplant populations, the frequency of IA observed in PT recipients appears relatively lower. IA has been reported in 0.7% to 4% of renal transplant recipients and 1% to 9.2% of liver transplant recipients, with a substantially higher risk in the setting of liver re-transplantation or concomitant renal failure. Lung transplant recipients demonstrate the highest incidence, ranging from 4% to 23%, likely related to continuous environmental exposure of the transplanted lung and higher immunosuppressive burden [6]. These differences highlight the heterogeneity of IA risk across transplant types and suggest that, although clinically significant, the overall burden in PT recipients is comparatively lower than in several other solid organ transplant populations.

Our findings demonstrate that IA-related hospitalizations among PT recipients more frequently occurred in patients with a lower overall comorbidity burden. We found that, compared with PT admissions without IA, those with IA had a significantly lower proportion of high comorbidity burden, defined as a CCI > 3 (54% vs. 68.6%). This observation suggests that these admissions were largely driven by acute infectious complications rather than by an extensive history of chronic disease. Similar patterns have been described previously, where IA has been characterized as a rapidly progressive opportunistic infection that can develop in patients with relatively limited baseline comorbidities following recent immune compromise. Such immune compromise may arise from immunosuppressive therapy, perioperative stress, or healthcare-associated exposures [2]. These observations highlight important limitations of traditional comorbidity indices, which are designed to capture chronic disease burden and may inadequately reflect short-term susceptibility to severe acute infections in transplant populations. As a result, reliance on baseline comorbidity scores such as CCI alone may underestimate the risk of serious infectious complications such as IA.

Pulmonary comorbidities, including bronchiectasis and chronic pulmonary disease, were more common among PT admissions complicated by IA. This association has been consistently observed in other high-risk populations with aspergillosis. Given that the lungs serve as the primary portal of entry for *Aspergillus* species, underlying structural lung disease and chronic inflammatory disease likely increase susceptibility to invasive fungal infection and subsequent hematogenous dissemination [10,11]. ARF occurred more than twice as frequently in IA-related hospitalizations, reflecting the severe pulmonary involvement commonly described in IA [12,13]. These observations emphasize the importance of close respiratory monitoring and early diagnostic consideration in PT recipients who develop respiratory symptoms in the post-transplant period.

The requirement for IMV was markedly higher in PT admissions complicated by IA, occurring more than four times as frequently compared with non-IA PT hospitalizations. This finding aligns with prior studies in SOT populations demonstrating frequent respiratory compromise necessitating ventilatory support in the setting of invasive fungal infections [14,15]. Such findings likely reflect delayed diagnosis, rapid disease progression, and the severity of pulmonary involvement in this population. A study conducted between 2008 and 2016 in SOT recipients identified the use of vasoactive drugs as an independent predictor of mortality but did not find IMV to be a significant factor [14]. In contrast, in our analysis focusing on PT recipients, IMV emerged as an independent predictor of all-cause in-hospital mortality among PT admissions complicated by IA. This finding underscores the prognostic significance of respiratory failure requiring mechanical ventilation and suggests that progression to ventilatory support represents a critical inflection point in disease severity and clinical course.

The high rates of sepsis, AKI, and ARF observed in the IA cohort further highlight the systemic and severe nature of this infection in these immunocompromised hosts. Prior studies have consistently linked IA-associated multiorgan dysfunction, particularly the development of AKI and ARF, with increased mortality in invasive fungal diseases [16,17]. In this analysis, all-cause hospital mortality was markedly higher among admissions complicated by IA, and these hospitalizations were characterized by nearly fourfold increases in hospital length of stay and overall healthcare costs. These findings are consistent with the critical and resource-intensive nature of IA-related illness [3,12]. The substantial economic burden likely reflects the need for prolonged intensive care, extensive diagnostic testing, targeted antifungal therapy, and management of multisystem complications. Longer hospital stays may also increase the risk of secondary infections and other hospital-related adverse events, further contributing to morbidity and delayed recovery, as reflected in the higher rates of non-routine discharge dispositions among IA admissions.

The poorer outcomes observed in IA admissions can be explained by the aggressive clinical course and rapid progression that are often seen with this disease, particularly in transplant recipients. However, it is notable that the outcomes observed in this PT population appear more favorable than those reported in recipients of other solid organ transplants, including liver, lung, kidney, and heart transplants [6]. Differences in transplant volume, recipient selection criteria, perioperative management, and post-transplant surveillance practices may also contribute to the comparatively more favorable outcomes observed in PT recipients. In addition, other SOT procedures have been performed more frequently and studied more extensively, resulting in more robust data and well-established standardized guidelines for infection prevention, monitoring, and management, which may influence reported outcomes across transplant populations.

Current clinical guidelines provide recommendations for *Candida* prophylaxis in PT recipients. However, there are no established protocols specifically addressing the prevention or management of *Aspergillus* infections in this group [6,7]. Limited evidence from single-center studies suggests that routine antifungal prophylaxis targeting *Aspergillus* may not be universally warranted. Such strategies could potentially complicate post-transplant care due to drug interactions with immunosuppressive therapies and concerns regarding toxicity or resistance [18,19]. In the present study, our dataset lacked detailed information on antifungal prophylaxis or treatment regimens, which prevented assessment of their use and impact on clinical outcomes. This limitation underscores the need for future studies with more granular clinical data to better inform preventive and therapeutic strategies for IA in PT recipients.

This study has several limitations, including the risk of misclassification bias inherent to administrative databases such as the NIS. Detailed clinical information, including the site, severity, and timing of IA, was not available, making it difficult to determine whether infection preceded or followed complications such as AKI or ARF. Diagnostic modalities commonly used to confirm IA, including imaging findings, galactomannan testing, and fungal cultures, are not captured in the database. In addition, the NIS does not provide clinical or microbiologic data to distinguish proven, probable, or possible cases of IA. It also lacks the information required to apply the European Organization for Research and Treatment of Cancer diagnostic criteria, including host factors indicative of immunocompromised status, which limits the ability to establish causal relationships between IA and observed complications.

Data on antifungal treatment regimens, immunosuppressive strategies, graft function, and graft survival were also unavailable, limiting insight into management decisions and long-term outcomes. Additionally, the structure of the NIS prevents tracking individual patients across multiple admissions, with each entry representing a separate hospitalization rather than a continuous patient history. This restriction limits longitudinal analysis and may obscure disease progression and treatment pathways [20]. Furthermore, the NIS captures hospital admissions rather than individual patients and lacks unique patient identifiers, which precludes calculation of patient-level incidence and restricts analyses to admission-based estimates.

Finally, the relative rarity of PT limited the ability to perform detailed subgroup analyses. Broader inclusion of other solid organ transplant populations and prospective, multicenter studies with granular clinical data are needed to better define risk factors, inform prevention strategies, and guide treatment approaches in this high-risk population.

## 5. Conclusions

This nationally representative analysis provides important insights into the burden of IA among PT recipients, a population for which data remain limited. Although the NIS lacks granular clinical detail regarding diagnostic modalities, antifungal therapy, immunosuppressive regimens, and timing of infection relative to transplantation, it enables large-scale evaluation of rare complications across hospitals at a national level in the United States. This broad sampling framework allows for assessment of real-world outcomes. While the overall frequency of IA was low, PT admissions complicated by IA had higher rates of AKI, ARF, venous thromboembolism, and sepsis. These admissions were also associated with prolonged length of stay, increased healthcare utilization, and markedly higher all-cause in-hospital mortality. In multivariable analysis, IMV use emerged as an independent predictor of all-cause in-hospital mortality among PT admissions complicated by IA. These findings highlight the need for prospective, multicenter research to define risk factors, optimize preventive strategies, and guide treatment decisions. In the meantime, increased clinical awareness, timely diagnosis, and appropriate antifungal use remain essential for improving outcomes in this vulnerable population.

## Figures and Tables

**Table 1:** Baseline characteristics of pancreatic transplant admissions with and without invasive aspergillosis.

Variable	Pancreatic Transplant Without IA (*n*: 65,730)	Pancreatic Transplant with IA (*n*: 250)	*p* Value
Age at admission in years, mean (±SD)	49.84 (7.89)	52.36 (9.8)	0.190
Age group (in years):			0.012
18–40	22.7%	18.0%	
41–60	59.0%	46.0%	
61–80	18.2%	36.0%	
>80	0.1%	0.0%	
Race:			0.676
White	68.0%	75.0%	
Black	17.8%	12.5%	
Hispanic	10.3%	10.4%	
Others ^†^	3.9%	*	
Female	46.6%	36.0%	0.132
Median household income:			0.837
Quartile 1 (poorest)	25.4%	30.0%	
Quartile 2	27.3%	26.0%	
Quartile 3	26.6%	22.0%	
Quartile 4 (wealthiest)	20.7%	22.0%	
Primary expected payer:			0.235
Medicare	63.4%	70.0%	
Medicaid	6.5%	0.0%	
Private Insurance	28.2%	30.0%	
Self-pay and other ^‡^	1.9%	0.0%	
Admitted to the teaching hospital	48.9%	46.0%	0.683
Charlson comorbidity index:			<0.001
0–1	5.6%	20.0%	
2–3	25.9%	26.0%	
>3	68.6%	54.0%	
Comorbidities:			
Bronchiectasis	0.3%	4%	<0.001
Cerebrovascular disease	5.2%	8.0%	0.366
Chronic obstructive pulmonary disease	5.0%	18%	<0.001
Coronary artery disease	30.4%	20.0%	0.106
Dyslipidemia	40.5%	30.0%	0.128
Tobacco use disorder	25.9%	38.0%	0.052
Hypertension	80.2%	72.0%	0.140
Obesity	7.5%	6.0%	0.688
Venous thromboembolism ^§^	6.0%	20.0%	<0.001
Complications:			
Acute kidney injury	36.7%	60.0%	<0.001
Acute respiratory failure	9.4%	28.0%	<0.001
Sepsis/Infection	46.1%	100.0%	<0.001
Procedure:			
Invasive mechanical ventilation	4.0%	18.0%	<0.001
Length of stay (in days):			<0.001
≤10	87.4%	46.0%	
11–20	9.2%	24.0%	
≥21	3.4%	30.0%	
Disposition of Patient:			<0.001
Routine	67.8%	40.9%	
Transfer to a short-term hospital	3.6%	9.1%	
Transfer to a facility ^‖^	9.6%	25.0%	
Home health care	17.7%	22.7%	
Left against medical advice	1.3%	*	

* ≤10 admissions cannot be reported due to database policy. ^†^: Includes Asian or Pacific Islanders, Native Americans, and others. ^‡^: Includes no charge, workers’ compensation, CHAMPUS, CHAMPVA, Title V, and other government programs. ^§^: Includes both pulmonary embolism and deep vein thrombosis. ^‖^ Includes Skilled Nursing Facility (SNF), Intermediate Care Facility (ICF), and other types of facilities.

**Table 2:** Factors associated with mortality in pancreatic transplant recipients with invasive aspergillosis hospitalizations on multivariable logistic regression.

Variable	Adjusted Odds Ratio	Lower Limit 95% Confidence Interval	Upper Limit 95% Confidence Interval	*p* Value
Female	2.213	0.260	18.828	0.467
Race				
White	Reference			
Black	3.596	0.293	44.120	0.317
Hispanic	5.238	0.285	96.315	0.265
Age	1.055	0.997	1.117	0.062
Bronchiectasis	17.042	0.891	325.788	0.060
Invasive Mechanical Ventilation	48.777	2.588	919.360	0.009

**Table 3:** Outcomes of pancreatic transplant admissions with and without invasive aspergillosis.

Variable	Pancreatic Transplant Without IA (*n*: 65,730)	Pancreatic Transplant with IA (*n*: 250)	*p* Value
Outcomes:			
All causes of patient mortality	2.0%	12.0%	<0.001
Length of stay in days, mean (±SD)	6.13 (5.89)	24.86 (27.1)	<0.001
Total hospital charges in US dollars, mean (±SD)	$94,938.73 ($117,465.4)	$378,494.1 ($494,343.2)	0.005
Total hospital costs in US dollars, mean (±SD)	$24,463.61 ($26,796.83)	$93,019.01 ($98,598.74)	0.023

## Data Availability

https://hcup-us.ahrq.gov/db/nation/nis/nisdbdocumentation.jsp (accessed on 10 March 2026).

## Abbreviations

The following abbreviations are used in this manuscript:

IA	Invasive Aspergillosis
SOT	Solid Organ Transplant
PT	Pancreas Transplant
NIS	National Inpatient Sample
HCUP	Healthcare Cost and Utilization Project
ICD 10 CM	International Classification of Diseases, Tenth Revision, Clinical Modification
ICD 10 PCS	International Classification of Diseases, Tenth Revision, Procedure Coding System
AKI	Acute Kidney Injury
ARF	Acute Respiratory Failure
IMV	Invasive Mechanical Ventilation
CCI	Charlson Comorbidity Index
